# SEC31a‐ATG9a Interaction Mediates the Recruitment of COPII Vesicles for Autophagosome Formation

**DOI:** 10.1002/advs.202405127

**Published:** 2024-10-03

**Authors:** Jiaming Nie, Shaoyang Ma, Linyue Wu, Ye Li, Jiao Cao, Meng Li, Peter Mei, Paul R. Cooper, Ang Li, Dandan Pei

**Affiliations:** ^1^ Key Laboratory of Shaanxi Province for Craniofacial Precision Medicine Research College of Stomatology Xi'an Jiaotong University Xi'an Shaanxi 710004 China; ^2^ Department of Oral Sciences Faculty of Dentistry University of Otago Dunedin 9016 New Zealand

**Keywords:** ATG9a, autophagosome formations, COPII vesicles, osteogenesis, SEC31a

## Abstract

Autophagy plays an important role in determining stem‐cell differentiation. During the osteogenic differentiation of mesenchymal stem cells (MSCs), autophagosome formation is upregulated but the reason is unknown. A long‐standing quest in the autophagy field is to find the membrane origin of autophagosomes. In this study, cytoplasmic coat protein complex II (COPII) vesicles, endoplasmic reticulum‐derived vesicles responsible for the transport of storage proteins to the Golgi, are demonstrated to be a critical source of osteoblastic autophagosomal membrane. A significant correlation between the number of COPII vesicle and the autophagy level is identified in the rat bone tissues. Disruption of COPII vesicles restrained osteogenesis and decreased the number and size of autophagosomes. SEC31a (an outer coat protein of COPII vesicle) is found to be vital to regulate COPII vesicle‐dependent autophagosome formation via interacting with ATG9a of autophagosomal seed vesicles. The interference of *Sec31a* inhibited autophagosome formation and osteogenesis in vitro and in vivo. These results identified a novel mechanism of autophagosome formation in osteogenic differentiation of stem cells and identified SEC31a as a critical protein that mediates the interplay between COPII and ATG9a vesicles. These findings broaden the understanding of the regulatory mechanism in the osteogenic differentiation of MSCs.

## Graphical abstract

During osteogenic differentiation of MSCs, autophagosome formation is upregulated but the reason is unknown. Here, COPII vesicles are demonstrated to be a critical source of osteoblastic autophagosomal membrane. The interact between SEC31a (COPII vesicles coat protein) and ATG9a regulates the formation of autophagosome. These findings broaden the understanding of the regulatory mechanism in osteogenic differentiation of MSCs.

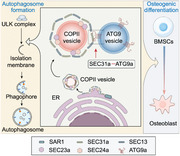

## 1 Introduction

The osteogenic differentiation of mesenchymal stem cells (MSCs) is vital for the maintenance of bone homeostasis, and it is now understood to be sophisticatedly regulated by macroautophagy/autophagy.^[^ ^1^, ^2^ ^]^ Since the increased level of autophagy in osteogenic differentiation was first published in 2013,^[^ ^3^ ^]^ the knowledge of how autophagy during osteogenesis has developed. Osteoblastic autophagy is required for maintaining bone mass by balancing bone homeostasis^[^ ^4^, ^5^ ^]^ and a deficiency in autophagy genes causes osteogenesis inhibition and significant bone loss.^[^ ^1^, ^6^, ^7^ ^]^ In previous studies, we have reported that autophagosome formation is increased to transport mitochondrial amorphous calcium phosphate (ACP) precursors for osteogenesis.^[^ ^8^ ^]^ Although studies have preliminarily investigated the functions of several autophagy‐related signaling pathways and genes during osteogenesis,^[^ ^9^, ^10^, ^11^, ^12^, ^13^ ^]^ how autophagosome forms, the key process in autophagy, remains to be clearly elucidated during osteogenesis.

Autophagosome formation generally contains four main steps: initiation of autophagosomal formation, nucleation of autophagosomal vesicles, elongation of the autophagosomal membrane, and the recognition of cargos.^[^ ^14^ ^]^ Through these steps, autophagosomal seed vesicles (diameter range 30–60 nm^[^ ^15^, ^16^ ^]^), develop into the mature autophagosome with a diameter of 300–900 nm in yeast^[^ ^17^ ^]^ and 500–1500 nm in mammals.^[^ ^18^ ^]^ A recent study has confirmed that autophagy‐related (Atg) 9 vesicles act as seeds for autophagosomal‐membrane formation,^[^ ^19^ ^]^ which are derived from the Golgi and are regarded as cytoplasmic membrane reservoirs that shuttle between this location and the phagophore assembly site (PAS).^[^ ^20^, ^21^ ^]^ Atg9 vesicles regulate autophagosome formation by affecting autophagosome nucleation.^[^ ^16^, ^19^, ^22^, ^23^ ^]^ However, Atg9 vesicles do not become a membrane source, but facilitate the delivery of membrane sources for autophagosomes.^[^ ^22^ ^]^ They form a platform for the recruitment of the autophagy machinery (including the Atg2‐Atg18 lipid transfer complex and the class III phosphatidylinositol 3‐phosphate kinase complex 1 (PI3KC3‐C1)). Indeed, Atg9 vesicles obtain lipids from the endoplasmic reticulum (ER)^[^ ^19^ ^]^ or other vesicles, such as the cytoplasmic coat protein complex II (COPII) vesicles.^[^ ^15^ ^]^

The membrane source is critical for *de novo* autophagosome formation.^[^ ^24^ ^]^ It has been reported that both COPII vesicles and Atg9 vesicles are recruited to the PAS, most likely providing the primary membrane material necessary to nucleate the initial phagophore.^[^ ^19^ ^]^ COPII vesicles, which are comprised of small GTPase (Sar1) and COPII coat proteins, including Sec23, 24, 13, and 31, play a crucial role in protein sorting of the anterograde vesicular transport process in mammals.^[^ ^25^ ^]^ Recently, emerging evidence has reinforced the COPII vesicle's role in autophagosome formation.^[^ ^26^, ^27^, ^28^, ^29^ ^]^ COPII vesicles have been found to be a critical membrane source for the formation of autophagosomes by triggering LC3 lipidation.^[^ ^30^ ^]^ Upon starvation stimulation, the COPII machineries translocate from the ER exit sites (ERES) to the ER‐to‐Golgi intermediate compartment (ERGIC),^[^ ^30^ ^]^ and then relocate from ERGIC to the PAS to promote autophagosome formation.^[^ ^29^ ^]^ Under osteogenic conditions, however, there is limited knowledge regarding the role of COPII vesicles in autophagosome formation.

The effect of COPII vesicles in autophagosome formation is dependent upon coat proteins (the inner layer SEC23 and 24, the outer layer SEC13 and 31). In adipocytes during obesity, SEC13 interacts with Atg7, leading to the increased formation of autophagosomes.^[^ ^31^ ^]^ An additional study found that the interaction between Sec24 and Atg9 regulates the autophagosome number in *Saccharomyces cerevisiae* under starvation stimulation conditions.^[^ ^15^ ^]^ The above observations suggest that although the step of autophagosome formation is essentially the same for all autophagy‐related pathways, the regulatory mechanisms vary depending on the specific physiological conditions. Here, we report the interaction between SEC31a and ATG9a mediated the recruitment of COPII vesicles by Atg9 vesicles to form autophagosomes during the osteogenic differentiation of MSCs. Inhibition of *SEC31a* caused the suppression of autophagosome formation and osteogenesis. This work increases our knowledge of the osteogenic differentiation of MSCs and identifies new potential mechanisms involved in the recruitment of COPII vesicles by Atg9 vesicles.

## 2 Results

### 2.1 COPII Vesicle Numbers are Associated with the Autophagy Level During Osteogenesis

**Figure 1:**
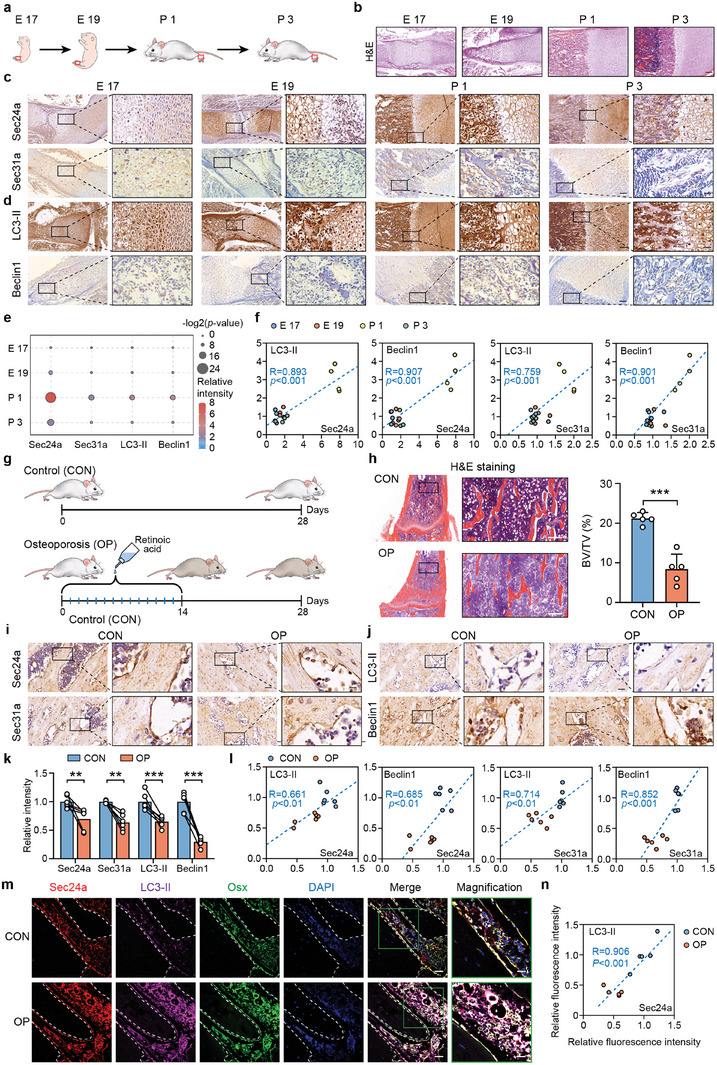
COPII vesicle amount is associated with autophagy level in rats bone tissue. a) Schematic representation of the rat development. The red boxes were used to map sample collection locations. E 17: embryonic day 17, E 19: embryonic day 19, P 1: postnatal day 1, P 3: postnatal day 3. b) Hematoxylin and eosin (H&E) staining of the right femur of rats at E17, E19, P1 and P3. Scale bars: 100 µm. c) Immunohistochemical staining of Sec24a and Sec31a of rat bone tissue. Scale bars: 50 µm and 20 µm, respectively. d) Immunohistochemical staining of LC3‐II and Beclin1 of rat bone tissue. Scale bars: 50 µm and 20 µm, respectively. e) Quantification of immunohistochemistry of Sec24a, Sec31a, LC3‐II and Beclin1 results. The color of the bubble represents immunohistochemical staining intensity and the size of the bubble represents the significance. f) The correlations between Sec24a‐LC3‐II, Sec24a‐Beclin1, Sec31a‐LC3‐II and Sec31a‐Beclin1 were indicated based on the relative immunohistochemical staining intensity. g) Schematic of the time course used for the in vivo experiments in rats with retinoic acid‐induced osteoporotic phenotypes (OP). h) H&E staining of rat right femur and quantitative analysis of bone volume/tissue volume (BV/TV). Scale bars: 50 µm. i) Immunohistochemical staining of Sec24a and Sec31a of bone tissue of the control group (CON) and the OP group. Black arrowheads represent Sec24a or Sec31a, respectively. Scale bars: 50 µm and 20 µm, respectively. j) Immunohistochemical staining of LC3‐II and Beclin1 of bone tissue of the CON group and the OP group. Black arrowheads represent LC3‐II or Beclin1, respectively. Scale bars: 50 µm and 20 µm, respectively. k) Quantification of immunohistochemistry of Sec24a, Sec31a, LC3‐II and Beclin1 results in the CON group and the OP group. l) The correlations between Sec24a‐LC3‐II, Sec24a‐Beclin1, Sec31a‐LC3‐II and Sec31a‐Beclin1 were indicated based on the relative immunohistochemical staining intensity of the CON group and the OP group. m) Three‐color immunofluorescence staining for Sec24a (Red), Osx (Green) and LC3‐II (Purple) expression of femurs from rats with or without osteoporosis. n) The correlation between Sec24a‐LC3‐II was indicated based on the immunofluorescence staining intensity. Scale bar: 20 µm (left) and 10 µm (right). ^**^ *P* < 0.01; ^***^ *P* < 0.001.

We initially confirmed the relevance of COPII vesicle levels in autophagy during osteogenesis. We collected femurs from rats at embryonic day 17 (E17), embryonic day 19 (E19), postnatal day 1 (P1) and postnatal day 3 (P3) (**Figure** **1**a), and examined bone development by H&E and Safranin‐O/fast green staining (Figure 1b; Figure S1, Supporting Information). The components of the inner and outer layer of the COPII vesicle (Sec24a and Sec31a) and autophagy markers (LC3‐II and Beclin1) were detected by immunohistochemistry (Figure 1c,d). Data showed that the expressions of these proteins increased during development, and peaked on P1 (Figure 1e; Figure S2, Supporting Information). Possible correlations between COPII vesicle proteins and autophagy markers were evaluated by calculating a Pearson correlation coefficient. The results showed that COPII vesicle and autophagy levels exhibited strong positive correlations (Figure 1f). We additionally used the three‐color immunofluorescence staining to detect the expression of Sec24a and LC3‐II in rat femurs. The results showed a similar expression trend and a strong correlation between Sec24a and LC3‐II in Osx+ cells (Figure S3, Supporting Information).

We utilized an osteoporosis rat model by feeding animals with retinoic acid for 14 days. This is a widely used and well characterized animal model which is used to imitate osteoporosis in humans (Figure 1g). The result of H&E staining showed the decrease in trabecular bone volume density (BV/TV), which confirmed the successful development of the rat model (Figure 1h). Subsequently, we detected the expression of Sec24a, Sec31a, LC3‐II, and Beclin1 using immunohistochemistry (Figure 1i,j), and identified that these proteins decreased during osteoporosis in rats (Figure 1k). The markers of COPII vesicles were associated with markers of autophagy (Figure 1l). Further, the three‐color immunofluorescence staining results of Sec24a, Osx and LC3‐II confirmed again the regulatory relationship between COPII vesicles and autophagy level (Figure 1m,n; Figure S4, Supporting Information).

### 2.2 Disruption of COPII Vesicle Impairs Osteogenesis

**Figure 2:**
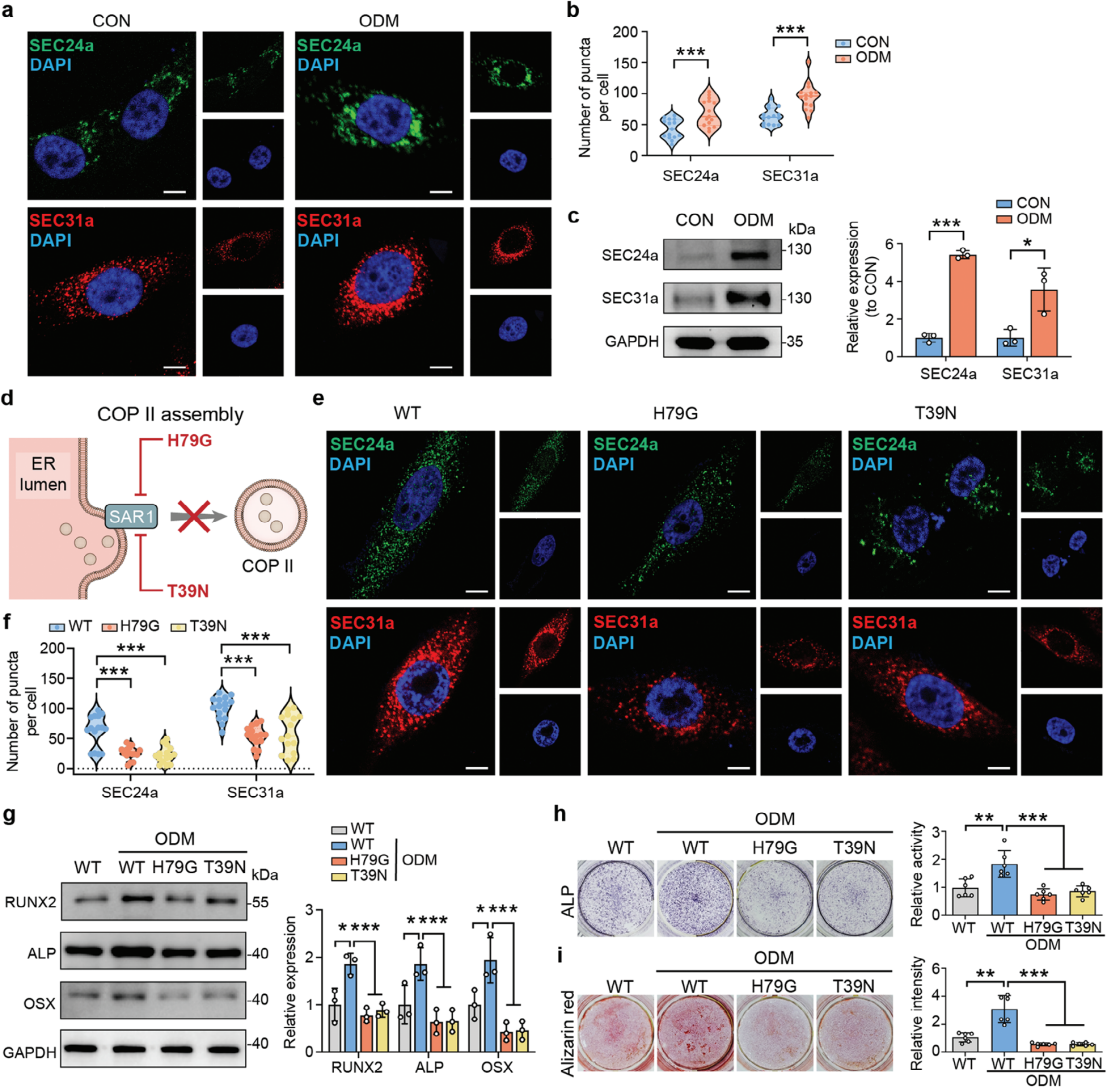
Disruption of COPII vesicle inhibits mineralization of BMSCs. a) Immunofluorescence of SEC24a and SEC31a of BMSCs in osteogenic differentiation medium (ODM). Scale bars: 20 µm. b) Quantification of the immunofluorescence signals of SEC24a and SEC31a from BMSCs in ODM. c) WB analysis and quantification showed the effect of osteogenic‐induction on the expression of SEC24a and SEC31a in BMSCs. d) Schematic representation of the disruption of COPII vesicles. e) Immunofluorescence of SEC24a and SEC31a of BMSCs transfected with H79G or T39N vector. Scale bars: 20 µm. f) Quantification of the immunofluorescence signals of SEC24a and SEC31a from BMSCs transfected with H79G or T39N vector. g) WB analysis and quantification showed the effect of COPII vesicle disruption on the expression of RUNX2, ALP and OSX. h) ALP staining and quantitative determination of ALP expression in BMSCs transfected with H79G or T39N vector for 7 days. i) Representative images and quantification of alizarin red staining showed the degree of alizarin red staining for mineral deposition on 21 days. ^*^ *P* < 0.05; ^**^ *P* < 0.01; ^***^ *P* < 0.001.

To explore the effect of the COPII vesicle on osteogenesis, we detected the amount of COPII vesicles in human bone marrow mesenchymal stromal cells (BMSCs) under osteoblast differentiation medium (ODM)‐induced conditions by immunofluorescence staining for SEC24a and SEC31a. Data demonstrated that the number of SEC24a and SEC31a puncta significantly increased during osteogenesis (**Figure** **2**a,b). Additionally, the protein expressions of SEC24a and SEC31a were upregulated under ODM‐induced conditions (Figure 2c). Subsequently, we developed COPII vesicle deficient cells by mutating *SAR1* at H79G or T39N (Figure 2d), as has been performed in previous studies.^[^ ^32^, ^33^, ^34^ ^]^ The expressions of SEC24a and SEC31a were notably decreased in cells transfected with the H79G or T39N vector construct (Figure 2e,f; Figure S5, Supporting Information), which showed that COPII vesicle assembly was successfully disrupted.

The osteogenic differentiation capacity of cells with H79G or T39N vector was then analyzed. The expressions of osteogenesis‐related proteins (RUNX2, ALP, and OSX) were downregulated by the disruption of COPII vesicles (Figure 2g; Figure S6, Supporting Information). The results of ALP staining (day 7) and alizarin red staining (day 21) also confirmed the impaired differentiation capacity (Figure 2h). In addition, ACP precursors, a marker of osteogenesis,^[^ ^8^ ^]^ were decreased in H79G and T39N mutant cells (Figure S7, Supporting Information). These data confirmed the regulatory function of COPII vesicles in osteogenesis, and indicated their potential involvement in autophagy.

### 2.3 Increase in Autophagosome Formation During Osteogenesis was Depended on COPII Vesicles

**Figure 3:**
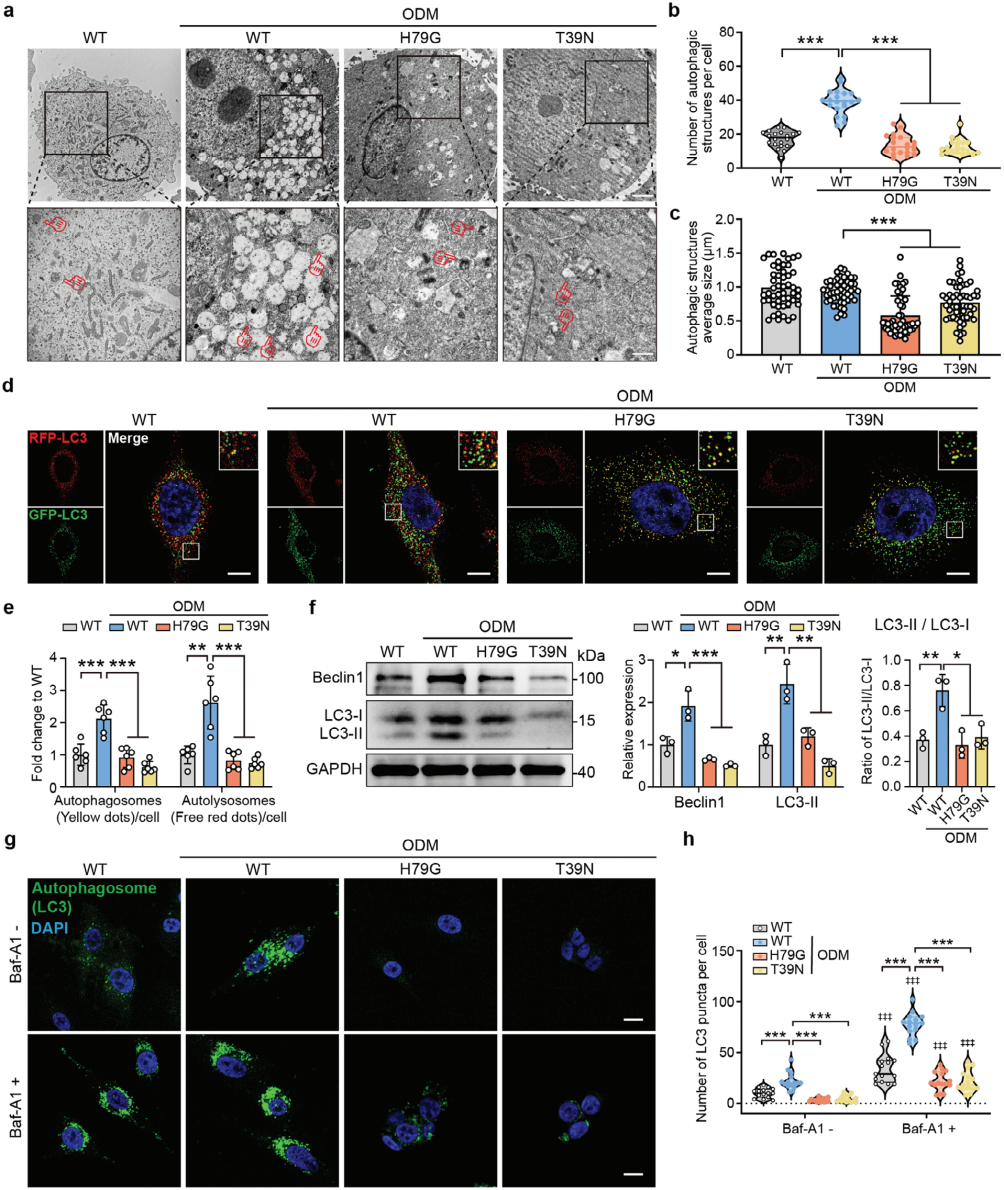
Disruption of COPII vesicle impairs autophagosome formation of BMSCs. a) The number of autophagosomes in the BMSCs transfected with H79G or T39N vector was detected by TEM. Scale bars: 2 µm. Red fingers illustrated typical autophagosomes. Scale bars: 1 µm. b) Quantification of the numbers of autophagic structures in BMSCs transfected with H79G or T39N vector under osteogenic condition. c) Quantification of average size of autophagic structure in BMSCs transfected with H79G or T39N vector under osteogenic condition. d) Immunofluorescence microscopy was performed to detect GFP‐LC3 and RFP‐LC3 puncta in BMSCs transfected with H79G or T39N vector under osteogenic condition. Scale bars: 10 µm. e) Quantification of autophagosomes (yellow dots) and autolysosomes (free red dots) in BMSCs transfected with H79G or T39N vector under osteogenic condition. f) WB analysis and quantification showed the effect of COPII vesicle disruption on the expression of BECLIN1 and LC3‐II. The ratio between LC3‐II and LC3‐I was calculated. g) Immunofluorescence staining for LC3 in BMSCs transfected with H79G or T39N vector under osteogenic condition with or without Baf‐A1. Scale bars: 20 µm. h) Quantification of LC3 puncta in BMSCs transfected with H79G or T39N vector under osteogenic condition with or without Baf‐A1. *** *P* < 0.001. ‡‡‡ *P* < 0.001 to Baf‐A1‐.

As autophagy participated in the regulation of osteogenesis,^[^ ^8^, ^35^ ^]^ and the level of autophagy exhibited strong positive correlations with the amount of COPII vesicles (Figure 1f,i), the autophagy levels in cells transfected with H79G or T39N vector were analyzed. The number of autophagosomes increased in wild‐type (WT) cells under osteogenic conditions (**Figure** **3**a,b). The disruption of COPII vesicles significantly decreased the number of autophagic structures (Figure 3a,b) as well as their average size (Figure 3a,c). To further evaluate autophagy activity after COPII vesicle disruption, we assessed autophagosomes and autolysosomes (lysosome fusion) with mRFP‐GFP tandem fluorescent‐tagged LC3.^[^ ^36^ ^]^ In the acidic lysosome environment, RFP‐LC3 was stable, while GFP‐LC3 was degraded. As shown in Figure 3d,e, osteogenic induction increased GFP‐ and RFP‐LC3 puncta (yellow dots), while COPII vesicle disruption attenuated this increase. In addition, the expression of LC3‐II and BECLIN1 significantly decreased in H79G or T39N vector‐transfected cells, as well as the ratio of LC3‐II:LC3‐I (Figure 3f). We further assessed the autophagy flux by treating the WT and COPII vesicle‐mutated cells with Bafilomycin A1 (Baf‐A1), an autolysosome inhibitor.^[^ ^37^ ^]^ The autophagosome numbers in cells transfected with H79G or T39N were substantially reduced even under Baf‐A1 treated conditions (Figure 3g,h). The above results suggested that autophagosome formation was inhibited by COPII vesicle disruption.

As nutrient starvation is the most understood form of autophagy induction, we detected the amount of COPII vesicles present under starvation conditions. Data showed that serum starvation increased the amount of COPII vesicles (Figure S8, Supporting Information) and autophagosomes (Figure S9, Supporting Information). However, the size of autophagosomes didn't change significantly under nutrient starvation (Figure S9, Supporting Information). The disruption of COPII vesicles decreased the number and average size of autophagosomes (Figure S9, Supporting Information). These data further demonstrated the function of COPII vesicles in autophagosome formation.

### 2.4 COPII Vesicles Function at the Early Stage of Autophagosome Formation

**Figure 4:**
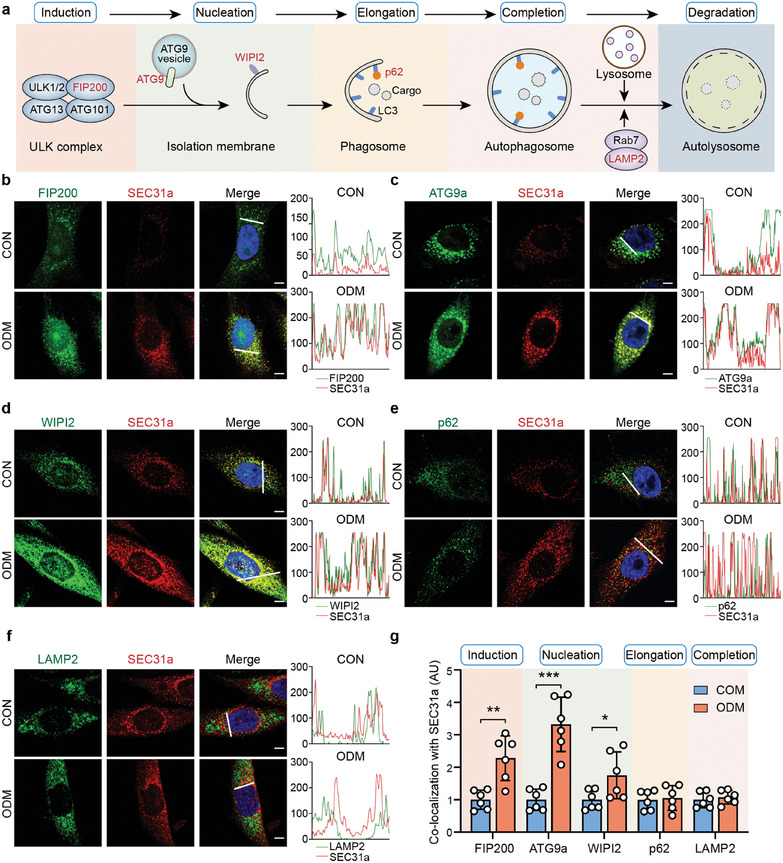
COPII vesicles function at the early stage of autophagosome formation. a) Schematic representation of the autophagy process. b‐f) Fluorescence confocal microscopy revealed the co‐localization of SEC31a and FIP200 b), ATG9a c), WIPI2 d), p62 e) and LAMP2 f) in BMSCs under osteogenic induction. Fluorescence intensity profiles along the white lines were showed on the right. Scale bars: 10 µm. g) Quantitation of SEC31a and autophagy‐related proteins co‐localization in BMSCs under osteogenic induction. ^*^ *P* < 0.05, ^**^ *P* < 0.01, ^***^ *P* < 0.001.

Autophagosome formation includes several steps: induction, nucleation elongation, and completion.^[^ ^38^ ^]^ To identify at which stage COPII vesicles function in autophagosome formation, we examined the colocalization of SEC31a and several biomarkers of autophagosome formation, including FIP200, ATG9, WIPI2, p62, and LAMP2 (**Figure** **4**a). The results of immunofluorescence staining showed that the colocalization of SEC31a and biomarkers of the early stages of autophagosome formation (including FIP200, ATG9, and WIPI2) increased substantially under osteogenic conditions (Figure 4b–d,g). In contrast, the colocalization of SEC31a and p62 or LAMP2 showed no significant changes after osteogenic induction (Figure 4e–g). To exclude the possibility that the changes in colocalizations were due to the expression changes of biomarkers, we performed immunofluorescence staining. Results showed that the expression of FIP200, ATG9a, WIPI2, and LAMP2 were significantly upregulated after osteogenic induction, whereas the level of p62 decreased (Figure S10, Supporting Information). These results indicated that COPII vesicles regulated the early stage of autophagosome formation.

### 2.5 COPII Vesicles Were Recruited to Atg9 Vesicles Through SEC31a‐ATG9a Interaction

**Figure 5:**
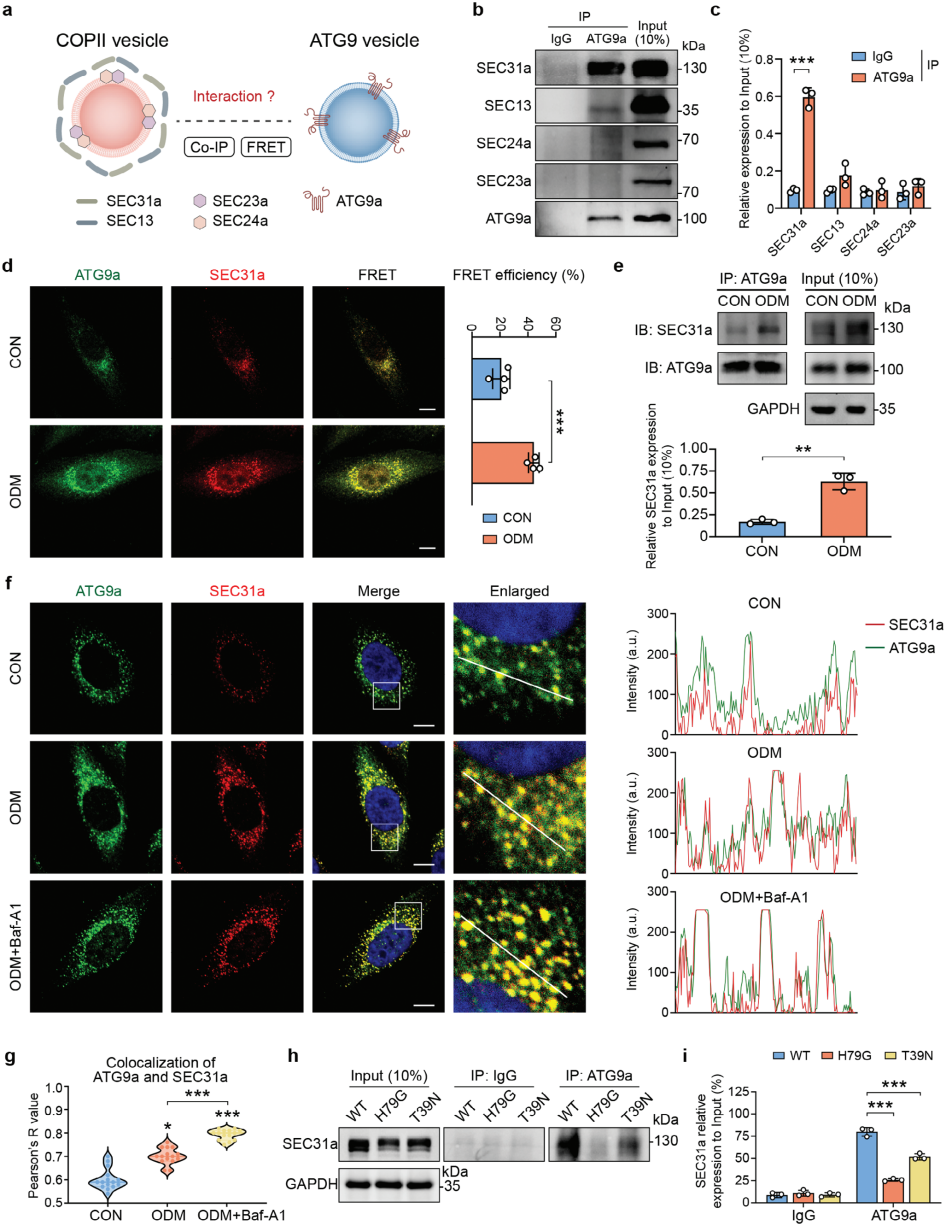
SEC31a interacts with ATG9a to regulate the autophagosome formation in BMSCs under osteogenic induction. a) Schematic illustration of experiment design. b) Co‐immunoprecipitation (Co‐IP) assay of the interaction between ATG9a and the coat proteins (SEC31a, SEC13, SEC24a and SEC23a) of COPII vesicles. Total proteins were immunoprecipitated with anti‐ATG9a beads. Input and IP proteins were analyzed by protein gel blot analysis with anti‐SEC31a, anti‐SEC13, anti‐SEC24a, anti‐SEC23a and anti‐ATG9a. c) Quantitation of COPII vesicles coat proteins interacted with ATG9a. d) Fluorescence resonance energy transfer (FRET) analyses. BMSCs with or without osteogenic induction treatment (ODM) were transfected with vectors encoding mClover‐ATG9a and mRuby‐SEC31a. The fluorescence images of mClover‐ATG9a (green), mRuby‐SEC31a (red), and merged images are shown. Scale bars: 10 µm. The efficiencies of FRET between the pair of ATG9a and SEC31a were analyzed. e) Co‐IP assay showing the interaction between SEC31a and ATG9a in BMSCs with or without osteogenic induction treatment (ODM). f) Fluorescence confocal microscopy revealed the co‐localization of SEC31a and ATG9a of BMSCs under osteogenic induction with or without Baf‐A1. Fluorescence intensity profiles along the white lines were showed on the right. Scale bars: 10 µm. g) The Pearson correlation was calculated for merged images to indicate the co‐localization between SEC31a and ATG9a. h) Co‐IP assay showed the effect of disruption of COPII vesicles on the interaction between SEC31a and ATG9a. i) Quantitation of the interaction between SEC31a and ATG9a in BMSCs transfected with H79G or T39N vectors. ^*^ *P* < 0.05, ^**^ *P* < 0.01, ^***^ *P* < 0.001.

Atg9 vesicles act as seeds of autophagosomes.^[^ ^19^ ^]^ A previous study showed that coat proteins assist COPII vesicles to form autophagosomes by interacting with Atg9 vesicles.^[^ ^15^ ^]^ We therefore detected the interaction between ATG9a and coat proteins by co‐immunoprecipitation (Co‐IP, **Figure** **5**a). Co‐IP results indicated that ATG9a interacted with SEC31a in preference to other coat proteins of COPII vesicles (Figure 5b,c). Additionally, we utilized bioinformatic analysis to identify the protein‐protein interaction (PPI) network for autophagosome formation‐related factors to identify the key molecules involved. The cytoHubba analysis demonstrated that ATG9a was central to autophagosome formation (Figure S11, Supporting Information). We also analyzed the publicly available data from the GEO database (https://www.ncbi.nlm.nih.gov/geo/). The results showed that SEC31a was the only coat protein with increased expression in all 6 datasets (Figure S12, Supporting Information). Subsequently, the interaction of SEC31a and ATG9a was further corroborated by fluorescence resonance energy transfer (FRET) and Co‐IP. The results showed that the expression of SEC31a and the colocalization of ATG9a and SEC31a increased in BMSCs under osteogenic induction (Figure 5d,e). To further confirm this interaction, SEC31a and ATG9a were respectively tagged with FLAG or HA tag, and detected by western blot. Results showed that FLAG‐tagged SEC31a could precipitate ATG9a and vice versa (Figure S13, Supporting Information). Additionally, the results of the immunofluorescent staining also confirmed the increased colocalization of ATG9a and SEC31a during osteogenesis (Figure 5f,g). Baf‐A1 was then used to accumulate autophagosomes by blocking autolysosome degradation, and this indicated that the interaction between ATG9a and SEC31a concomitantly increased (Figure 5f,g). Additionally, we found that the disruption of COPII vesicles not only decreased the expression of SEC31a but also inhibited the interaction between SEC31a and ATG9a (Figure 5h,i). Taken together, SEC31a interacts with ATG9a, and their interaction is enhanced upon osteogenic induction.

### 2.6 Interference of SEC31a Restrains Autophagy and Osteogenesis in BMSCs

**Figure 6:**
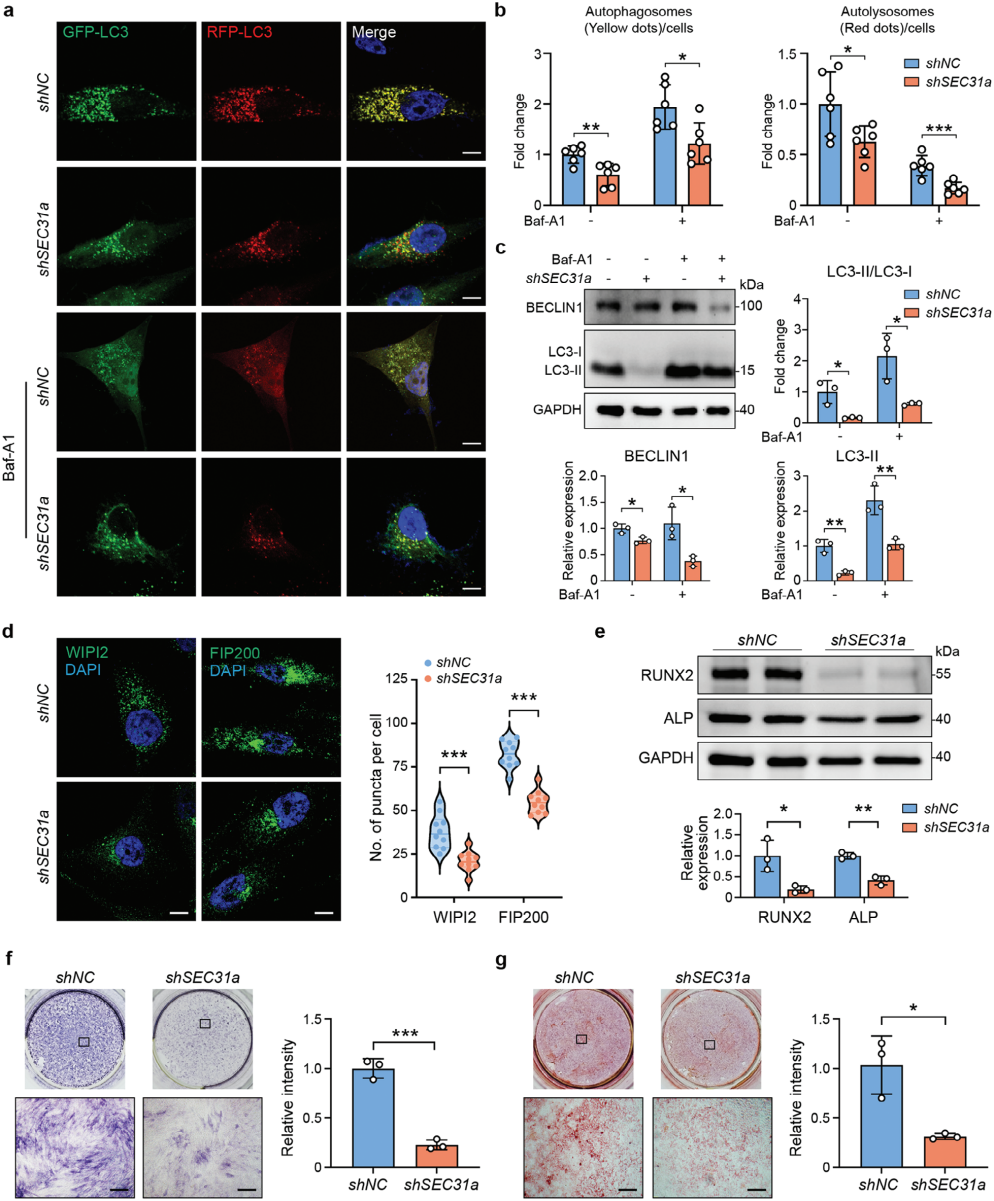
*ShSEC31a* restrains autophagy and osteogenesis in BMSCs. a) Immunofluorescence microscopy was performed to detect GFP‐LC3 and RFP‐LC3 puncta in BMSCs transfected with *shSEC31a* with or without Baf‐A1. Fluorescence intensity profiles along the white lines were showed on the right. Scale bars: 10 µm. b) Quantification of autophagosomes (yellow dots) and autolysosomes (free red dots) in BMSCs transfected with *shSEC31a* with or without Baf‐A1. c) WB analysis and quantification showed the effect of *shSEC31a* on the expression of BECLIN1 and LC3‐II, and the ratio of LC3‐II/LC3‐I. d) Immunofluorescence staining for WIPI2 and FIP200 and quantification of WIPI2 and FIP200 puncta in BMSCs transfected with *shSEC31a*. Scale bars: 20 µm. e) WB analysis and quantification showed the effect of *shSEC31a* on the expression of RUNX2 and ALP. f) ALP staining and quantitative determination of ALP expression in BMSCs transfected with *shSEC31a* for 7 days. g) Representative images and quantification of alizarin red staining showed the degree of alizarin red staining for mineral deposition on 21 days. ^*^ *P* < 0.05; ^**^ *P* < 0.01; ^***^ *P* < 0.001.

To determine the specific effect of SEC31a on autophagy and osteogenesis, we construct lentiviral shRNA specific for *SEC31a*. *ShSEC31a* transfection downregulated the expression of SEC31a in BMSCs with or without osteogenic induction (Figure S14, Supporting Information). We further examined autophagic flux using the RFP‐GFP‐LC3 assay. Representative images and quantification of GFP‐LC3 and RFP‐LC3 puncta showed significant decreases in autophagy in BMSCs transfected with *shSEC31a* even under Baf‐A1 treatment conditions (**Figure** **6**a,b). Additionally, the expressions of BECLIN1 and LC3‐II were downregulated by *shSEC31a*, as well as the ratio of LC3‐II:LC3‐I (Figure 6c). We also determined the expression of the early molecular markers of autophagy by immunofluorescence, and the data demonstrated that the expressions of WIPI2 and FIP200 were significantly decreased by *shSEC31a* (Figure 6d). Collectively, these results indicated that SEC31a, the coat protein of COPII vesicles, performs important functions in the regulation of autophagy.

As COPII vesicles regulated osteogenesis by affecting autophagosome formation, we then examined the role of SEC31a in osteogenesis. The *shSEC31a* transfection downregulated the expressions of several osteogenesis‐related genes (Figure 6e). Moreover, quantitative analysis of ALP activity (day 7, Figure 6f) and alizarin red S staining (day 21, Figure 6g) confirmed the role of SEC31a on osteogenesis.

### 2.7 Inhibition of Sec31a Suppresses Osteogenesis of Rat Bone Tissue

**Figure 7:**
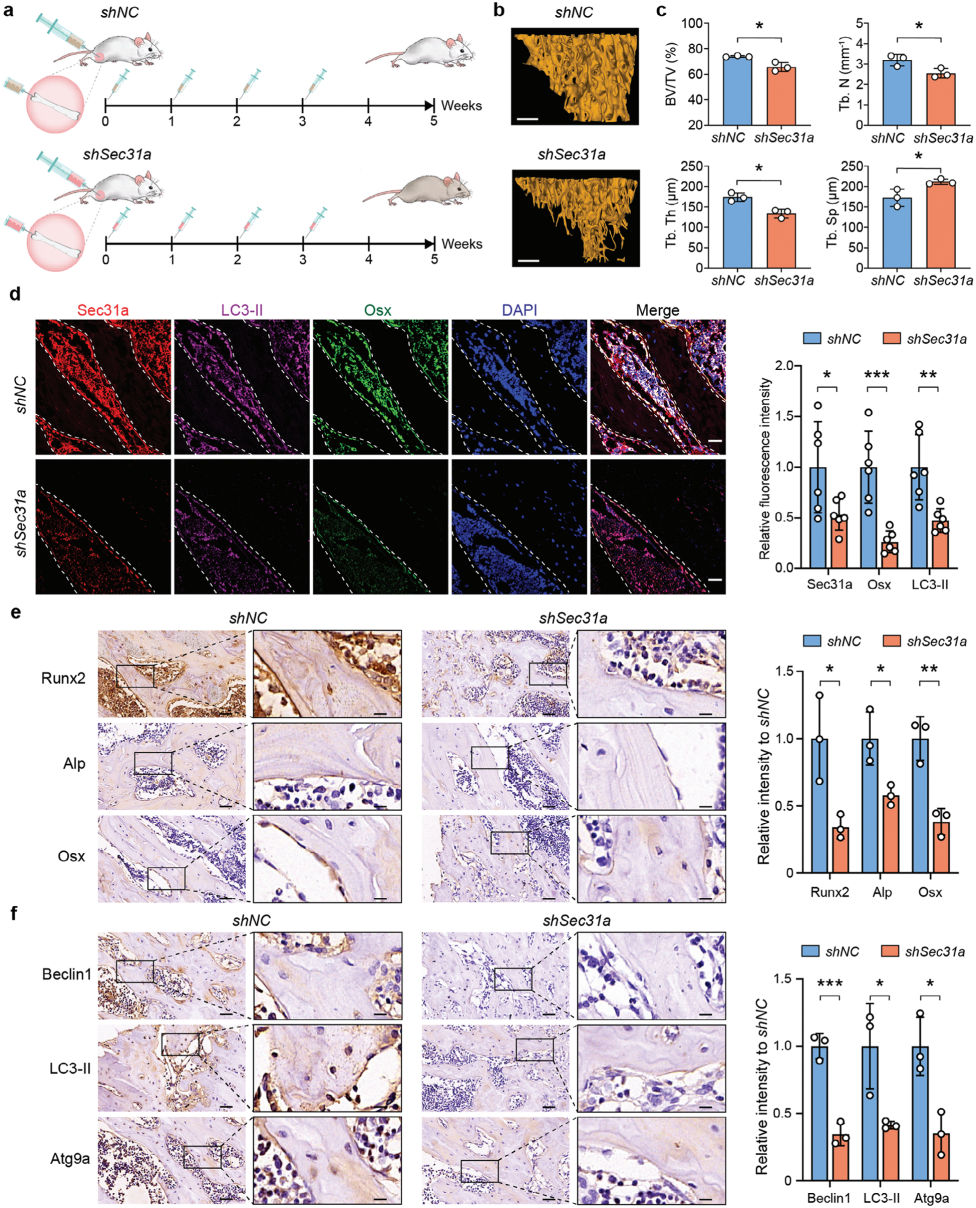
Injection of *shSec31a* adenovirus into bone marrow cavity suppresses the mineralization of rat bone tissue. a) Schematic illustration of experiment design. b) Representative micro‐CT 3D images. Scale bars: 0.5 mm. c) Quantitative analysis of BV/TV, trabecular number (Tb. N), trabecular thickness (Tb. Th) and trabecular separation (Tb. Sp). d) Three‐color immunofluorescence staining for Sec31a (Red), LC3‐II (Purple) and Osx (Green)in rat femur with *shNC* or *shSec31a* injection, and quantitative immunofluorescence analysis. Scale bar: 20 µm. e,f) Immunohistochemical staining of osteogenic‐related proteins (Runx2, Opn and Osx (e)) and autophagy‐related proteins (Beclin1, LC3‐II and Atg9 (f)). Quantitative analysis of immunohistochemical staining intensity was shown on the right. Scale bars: 50 and 20 µm, respectively. ^*^, *P*<0.05; ^**^, *P*<0.01; ^***^, *P*<0.001.

To confirm the relevance of the findings in cell culture to bone development in vivo, we evaluated the effect of inhibiting Sec31a expression in osteogenesis of rat bone tissue. The marrow cavities of the femur of young rats (< 4 months) were injected with the *shNC* or *shSec31a* lentivirus weekly for four consecutive weeks (**Figure** **7**a). Immunohistochemistry analysis confirmed that the injection of *shSec31a* reduced the expression of Sec31a (Figure S15, Supporting Information). We then analyzed the micro‐architectural properties of femurs using microcomputed tomography (micro‐CT). The results demonstrated that inhibition of *shSec31a* significantly decreased bone mass, as shown by BV/TV, reduced trabecular number (Tb.N), reduced trabecular thickness (Tb.Th), and increased trabecular separation (Tb.Sp) (Figure 7b,c). Subsequently, we performed H&E staining and Goldner staining to determine the changes in bone development patterns (Figure S16, Supporting Information). Compared with rats injected with *shNC*, those injected with *shSec31a* showed bone dysplasia represented by a lower ratio of bone area to total femur area (Figure S16, Supporting Information). Additionally, we performed immunofluorescence staining against Sec31a, Osx, and LC3‐II in rat femur with *shNC* or *shSec31a* injection. The results showed that the administration of *shSec31a* caused a significant impairment of Sec31a, LC3‐II, and Osx (Figure 7d). The results of immunohistochemistry also confirmed that *shSec31a* injection significantly inhibited the expression of osteogenic‐ and autophagy‐related genes in rat femur (Figure 7e,f). Overall, these results strongly support the relevance and alignment of the in vivo and in vitro findings.

## 3 Discussion

The increase of autophagosome formation has been observed and proven to promote osteogenesis in previous studies.^[^ ^8^ ^]^ However, the reasons accounting for the increase of autophagosome formation were unknown. Here for the first time, we have identified the regulatory function of COPII vesicle in autophagosome formation under osteogenic conditions. The disruption of COPII vesicles resulted in the inhibition of osteogenesis by decreasing autophagosome formation. Furthermore, we found SEC31a, the coat protein of COPII vesicles, mediated the formation of autophagosome by interacting with Atg9a of autophagosome seed vesicle. Ultimately, we demonstrated that inhibition of *Sec31a* leads to impaired osteogenesis in rats. Consequently, this study identified a novel regulatory mechanism for autophagosomes during osteogenesis, which involved COPII vesicles and its coat protein SEC31a.

Autophagy plays a vital role in bone homeostasis.^[^ ^5^, ^39^, ^40^, ^41^, ^42^ ^]^ Indeed, reduced autophagic activity appears to be likely a significant cause of age‐associated bone loss. Furthermore, dysfunction of autophagy‐related proteins, such as ATG5 and Beclin1, results in the loss of bone,^[^ ^7^, ^43^, ^44^ ^]^ whereas the activation of autophagy rescues bone metabolic disorders.^[^ ^39^ ^]^ In previous studies, we have found that autophagic vesicles may act as “the bridge” to transport ACP granules to the ECM for osteogenesis.^[^ ^8^ ^]^ A body of evidence has increased our understanding of autophagy‐dependent secretion,^[^ ^45^ ^]^ which contains cytokines, granule contents, and biomineral precursor ACP.^[^ ^8^, ^46^ ^]^ In this study, we confirmed that “the bridge” was removed with the mutation of COPII vesicles, which subsequently suppressed autophagy activity. Moreover, autophagic vesicles in osteoblasts are conducive to the ACP precursor cargo for osteogenesis. The intramitochondrial ACP granules appeared dispersive, while vast electron‐dense granules were found in autophagic vesicles in a compressive manner. These autophagic structures provide efficient carriers in osteogenesis. Thus, we concluded that autophagy plays a direct role in osteogenesis, and the complex molecular mechanism associating autophagy and osteogenesis requires further exploration.

The relationship between the autophagic process and cellular secretion is complex. COPII vesicles, derived from ER, typically mediate ER‐Golgi traffic for cargo delivery.^[^ ^47^ ^]^ Notably, COPII vesicles can also shift their role to forming autophagosomes in response to elevated levels of autophagy in yeast,^[^ ^15^ ^]^ adipocyte,^[^ ^31^ ^]^ and plant cell.^[^ ^48^ ^]^ COPII proteins can be recruited to the ERGIC and promote the levels of LC3 lipidation.^[^ ^30^, ^34^ ^]^ Furthermore, secretory factors, including COPII vesicle‐related proteins, SNAREs, and Tethers, are required for autophagy.^[^ ^49^, ^50^ ^]^ Here, we demonstrated that impairment of COPII vesicles decreased the number of autophagosomes in cells transfected with the H79G or T39N vectors (Figure 3b). Interestingly, the abnormality of the COPII vesicles also observably reduced the size of autophagosomes (Figure 3c). Our observations indicated that autophagosomes from H79G and T39N mutant cells were approximately half the size of those from control cells, and our data suggest that COPII vesicles may be the primary source of the autophagosome.

The source of autophagosomal membrane is a subject of intense current research and remains poorly understood. Currently, known sources of autophagosomes include ER,^[^ ^51^ ^]^ nuclear membrane,^[^ ^52^ ^]^ Golgi apparatus,^[^ ^53^ ^]^ mitochondrial,^[^ ^54^ ^]^ lipid droplets^[^ ^55^ ^]^ and COPII vesicles.^[^ ^56^ ^]^ In this study, we found the number and the size of autophagosomes were markedly reduced after disrupting the formation of COPII vesicles (Figure 3). Additionally, we observed the colocalization between SEC31a (the outer protein of COPII vesicle) and the biomarkers of the early stage of autophagosome formation (FIP200, ATG9a, and WIPI2) in Figure 4, which was increased by osteogenic induction. Based on these results, we claim that COPII vesicles are the primary membrane source for autophagosomes during osteogenic differentiation. Nevertheless, how other membrane sources regulate the formation of autophagosomes needs to be further explored.

COPII components play an essential role in the formation of autophagosomes.^[^ ^15^, ^26^, ^57^, ^58^, ^59^, ^60^ ^]^ COPII subunits SEC24ab and SEC23b can be phosphorylated by ULK1, and COPII vesicle can be relocated to ERGIC to facilitate the formation of autophagosomes under serum starvation conditions in mammalian cells.^[^ ^10^ ^]^ Notably, phosphorylated SEC24 intensifies the relationship with ATG9 and increases autophagosome numbers in yeast under nutrient‐deprived conditions.^[^ ^15^ ^]^ By dissecting the details of the COPII vesicle role on autophagy, it was initially identified that osteogenic induction reinforced the connection between ATG9 and COPII vesicle element SEC31 (Figure 5). COPII vesicle SEC13‐31 outer cage regulated vesicle size variation based on intrinsic flexibility.^[^ ^25^ ^]^ Thus, we speculated that SEC31 on autophagosomes may modify the size necessary to transport ACP efficiently. Our study suggests a novel function of COPII vesicle outer cage SEC31a, which would enrich the understanding of how, and for what purpose, autophagosome formation increases during osteogenesis.

## 4 Conclusion

To the best of our knowledge, this is the first report on the function of COPII vesicles in osteogenesis, which involves the regulation of autophagosome formation. SEC31a is responsible for COPII vesicle‐dependent autophagosome formation by interacting with ATG9a. Deficiency of SEC31a impairs autophagosome formation and osteogenic capacity. The SEC31a‐ATG9a interaction better clarifies the relationship between COPII vesicles and autophagosome formation in osteogenesis. Overall, these findings identify novel targets and approaches to therapeutically reverse autophagy‐related diseases.

## 5 Experimental Section

### Animals, Osteoporosis Model, Developmental Model, and Intra‐Bone Marrow Cavity Injection

For the osteoporosis model, female Sprague‐Dawley rats (*n* = 10, 9–10 weeks old) were obtained from the Animal Center of Xi'an Jiaotong University (Xi'an, China), and randomly divided into a control group (*n* = 5) and an osteoporosis group (*n* = 5). The rats for osteoporosis model group were administered 75 mg kg^−1^ body weight/day of retinoic acid (Solarbio, A9120) for two weeks via intragastric administration, while the control group was intragastrically administered with normal saline (0.9% NaCl) (Solarbio, IN9000).^[^ ^61^ ^]^ All rats were sacrificed after 2 weeks treatment. The femur and visceral organs were immediately collected from each rat for analysis in further studies.

For the developmental animal model, ten‐week‐old female and male Sprague–Dawley rats were purchased from the Animal Center of Xi'an Jiaotong University (Xi'an, China). Following mating, the observed plug date was denoted as E 0. The pregnant rats were sacrificed by an overdose of anesthetic before harvesting the embryos. In addition, the day when postnatal baby rats were born was denoted as P 1. The bones from the lower extremity of animals were immediately collected for further studies.

For intra‐bone marrow cavity injection, male Sprague–Dawley rats (*n* = 6, 9–10 weeks old) were purchased from the Animal Center of Xi'an Jiaotong University (Xi'an, China) and randomly assigned to two groups. All procedures were carried out as previously described.^[^ ^62^ ^]^ Briefly, Sprague‐Dawley rats were anesthetized with 3 mg mL^−1^ pentobarbital sodium. The area of tissue surrounding the knee joint was shaved and disinfected with iodophor. The knee was flexed to 90° and the proximal side of the femur was drawn to the anterior. A 28‐gauge needle was used to inject 5 × 10^9^ PUF/30 µL *shNC* or *shSec31a* adenovirus (Tsingke, China) into the bone marrow cavity of the femur.

All animal experiments were conducted under the ethical approval by the Biomedical Ethics Committee of Medical College, Xi'an Jiaotong University (No. 2021–1564). All animals were kept under 12 h light/12 h dark cycle conditions and ad libitum access to tap water and standard chow.

### Micro‐CT Analysis

Femurs from the sacrificed animals were collected and quickly fixed in 4% paraformaldehyde (Solarbio, P1110) at room temperature for 48 h and analyzed by high‐resolution micro‐CT (YXLON, Germany). All samples were scanned at 90 kV and 55.6 µA at 9 µm resolution. The 3D images and graphic data analysis were obtained via VG Studio MAX 3.0.2 (Volume Graphics, Germany) in accordance with the recommendations of the American Society for Bone and Mineral Research.^[^ ^63^ ^]^ BV/TV, Tb.N, Tb.Th and Tb.Sp was evaluated from the BioQuant Osteo analysis as previously described.^[^ ^64^ ^]^

### Histomorphological Analysis and Immunohistochemistry Staining

The bone samples were decalcified in 10% EDTA solution (Solarbio, E1171) for 28 days at 37 °C. The bone samples were dehydrated, embedded in paraffin, and dissected into 4.0 µm sections. For histomorphological analysis, H&E staining (Solarbio, T1300), Goldner staining (Solarbio, G3550), and Saffron‐O/fast Green Staining (Solarbio, G1371) were performed on the sections, and the images were analyzed using BioQuant Osteo software (Bioquant, USA) according to the manufacturer's instructions. For immunohistochemistry staining, antigen retrieval was performed by using enzyme complexes (Boster, AR0023) at room temperature for 30 min. The tissues were incubated with the primary antibody for 16 h and then incubated with the secondary antibody for 1 h at room temperature the following day. The samples were incubated with streptavidin‐biotin complex SABC‐AP (Boster, SA1020). A DAB (3,3′‐diaminobenzidine) color development kit (Boster, AR1027) was used to detect the protein level. The primary antibodies for Sec31a (Santa Cruz, sc‐376587), Sec24a (Affinity, DF12315), Beclin1 (ABclonal, A21695), LC3‐II (Proteintech, 14600‐1‐AP), Runx2 (ABclonal, A2851), Alp (Santa Cruz, sc‐365765), Osx (Santa Cruz, sc‐393325), and Atg9a (Abcam, ab108338) were used. All experiments were carried out using the manufacturers’ standard protocol. Captured images were analyzed using the ImageJ v1.8.0.

### Cell Culture

BMSCs (Procell, CP‐H166) cell line was purchased and maintained in Dulbecco's Modified Eagle's Medium (DMEM, Gibco, 11 965 092), supplemented with 10% fetal bovine serum (FBS, Gibco, 10 091 148) and 1% penicillin‐streptomycin (Beyotime, C0222). Cells were incubated in a 37 °C, 5% CO_2_ atmosphere. To induce osteogenesis, the cells were cultured in osteogenic differentiation medium (ODM) containing 10% FBS, 1% penicillin‐streptomycin, 50 µM ascorbic acid (Sigma–Aldrich, AX1775), 10 mM β‐glycerophosphate (Sigma–Aldrich, G9422), and 100 nM dexamethasone (Sigma–Aldrich, D4902).

### Western Blot Analysis

Cells were lysed using RIPA lysis buffer (Beyotime, P0013B) supplemented with a protease inhibitor cocktail (Beyotime, P1005) for 20 min. The protein concentration was quantified using a BCA protein assay kit (Beyotime, P0009). The cell extracts were denatured by adding 5 × SDS sample loading buffer (Beyotime, P0015) at 100 °C for 10 min. Supernatants were separated by 8–15% SDS–PAGE, and transferred to PVDF membranes (Merck Millipore, IPVH00005), incubated with primary antibodies at 4 °C overnight, and then with matched secondary antibodies at room temperature for 1 h. Immunoblots were visualized using an enhanced chemiluminescence substrate kit (Merck Millipore, C0712). ImageJ software v1.8.0 analysis was used to detect protein levels. The primary antibodies for SEC31a (Santa Cruz, sc‐376587), SEC24a (Abcam, ab262869), OSX (Santa Cruz, sc‐393325), RUNX2 (ABclonal, A2851), ALP (Santa Cruz, sc‐271431), BECLIN1 (ABclonal, A21695), LC3B (Proteintech, 14600‐1‐AP), ATG9a (Abcam, ab108338), GAPDH (Proteintech, 60004‐1‐Ig), FLAG (Proteintech, 66008‐4‐Ig), and HA (Proteintech, 51064‐2‐AP) were used. Target protein levels were normalized to those of the GAPDH housekeeping protein.

### Reverse Transcription‐Quantitative Polymerase Chain Reaction (RT‐qPCRs)

RT‐qPCRs were performed to compare mRNA expressions. Total RNA was isolated using the Takara mini BEST Universal RNA extraction kit (Takara, 9767). cDNA was prepared from total RNA by RT using oligo‐dT primers (Takara, 6110A), and qRT‐PCR was performed using SsoFast EvaGreen Super Mix (Bio‐Rad, 1 725 201) according to the manufacturer's instruction. A total reaction mixture of 20 µL was amplified in a 96‐well PCR plate (Bio‐Rad, HSP9601). Relative gene expression levels were calculated using 2^−ΔΔCt^ method.

qPCR primers used in this study:

Human *OSX* forward: 5′‐GGAAAGGAGGCACAAAGAAGC‐3′;
Human OSX reverse: 5′‐CCCCTTAGGCACTAGGAG‐3′;
Human ALP forward: 5′‐GACCTCCTCGGAAGACACTC‐3′;
Human ALP reverse: 5′‐TGAAGGGCTTCTTGTCTGTG‐3′;
Human RUNX2 forward: 5′‐ATTTAGGGCGCATTCCTCATC‐3′;
Human RUNX2 reverse: 5′‐TGTAATC TGACTCTGTCCTTGTGGAT‐3′;
Human SEC31a forward: 5′‐TAGAGCCCAGGCCAAAACTG‐3′;
Human SEC31a reverse: 5′‐AAACTCCAGTTACCAGCCCG‐3′;
Human GAPDH forward: 5′‐TCGACAGTCAGCCGCATCT‐3′;
Human GAPDH reverse: 5′‐CCGTTGACTCCGACCTTCA‐3′.

### Immunoprecipitation Assay

Cells were lysed on ice using NP‐40 lysis buffer (Beyotime, P0013F) for 15 min, and a protease inhibitor cocktail (Beyotime, P1005) was added. The cell extract was obtained by centrifugation at 12000 × g for 10 min at 4 °C. Following pretreatment with magnetic beads in a cradle at 4 °C for 30 min, the cell extracts (0.5 mg) were incubated with magnetic beads for 2 h, and then incubated with primary antibodies at 4 °C with rotation overnight. The primary antibodies used were ATG9a (Cell Signaling Technology, D4O9), FLAG (Proteintech, 66008‐4‐Ig), and HA (Proteintech, 51064‐2‐AP). The magnetic beads were retrieved using a magnetic shelf. The immunoprecipitates were washed five times with washing buffer and then denatured with 2 × SDS sample loading buffer (Beyotime, P0750) at 100 °C for 5 min. Subsequently, the samples were analyzed by western blot.

### Fluorescence Resonance Energy Transfer (FRET)

In FRET experiments, images of donor (Alexa Fluor 488 green) and acceptor (Alexa Fluor 568 red) fluorescence were acquired before and after photobleaching using an Olympus FluoView FV‐100 microscope driven by Olympus FV‐1000 3.1 software. The donor (Alexa Fluor 488 green, Abcam, ab150113), and acceptor (Alexa Fluor 568 red, Abcam, ab175470) were used for FRET experiments. The sample preparation was conducted in accordance with previous studies,^[^ ^65^ ^]^ and all images were captured from random fields of view.

### Transmission Electron Microscopy

Cells were harvested and processed for analysis. Initially, samples were fixed with 2.5% glutaraldehyde (Solarbio, P1126) in 0.1 m sodium cacodylate buffer at room temperature for 4 h. Subsequently, they were postfixed with 1% osmium tetroxide in 0.1 m sodium cacodylate buffer at room temperature for 45 min. Following this, the samples underwent gradient dehydration: 50% ethanol for 15 min, 70% ethanol for 15 min, 90% ethanol for 15 min, and finally 100% ethanol for 30 min. The dehydrated samples were then embedded in epoxy resin. Ultrathin sections (80–90 nm) were prepared and stained with uranyl acetate and Reynold's lead citrate. Imaging was performed using a transmission electron microscope operating at 80 kV (Hitachi, H‐7650B).

### ALP Staining and Alizarin Red S Staining

For ALP staining, osteogenesis‐inducing cells were fixed with 4% paraformaldehyde for 15 min at room temperature and subjected to staining with ALP staining solution (Beyotime, P0321S) in the dark. For alizarin Red S staining, the remaining cells were also treated with 4% paraformaldehyde and stained with Alizarin Red S staining solution (Sigma–Aldrich, A5533) for 10 min in a dark environment. Samples were destained using 10% (v/v) cetylpyridinium chloride monohydrate (Sigma–Aldrich, C0732) for 30 min, and absorbance values were obtained at 595 nm.

### Plasmid Construction and Transfection

SEC31a‐FLAG and ATG9‐HA plasmids (Tsingke, XA0049321) were constructed by inserting their sequences between the *EcoR*I and *Not*I sites of the pEGFP‐N1 vector. Additionally, Sar1a^H79G^ (referred to as H79G) mutant and Sar1a^T39N^ (referred to as T39N) mutant plasmids were generated between the *EcoR*I and *Xba*I sites of the plvx‐puro vector. *SEC31a*‐*RNAi* plasmids were purchased from Genechem (#001 34449) to inhibit the expression of *SEC31a* in hBMSCs. The lentiviral shRNA targeting rat *Sec31a* gene (Tsingke, China) was constructed for the femur bone marrow cavity injection. The LC3‐GFP and LC3‐mcherry plasmids were obtained from Vigene Biosciences (AD202001). The transfection of cells using these plasmids was carried out using Lipofectamine 3000 (Life Technologies, L3000150) for 18 h, following the manufacturer's instructions.

### Immunofluorescence Staining

Immunofluorescence staining was carried out following a previously described protocol.^[^ ^66^ ^]^ Briefly, the bone samples underwent the deparaffinization and rehydration, then were cooked with the antigen retrieval (BOSTER, AR0026) for 20 min at RT temperature, followed by cell permeabilization with 0.3% Triton X‐100 for 10 min. Then, the sections were immersed in 3% hydrogen peroxide for 15 min at RT temperature to block the endogenous catalase, and blocked with QuickBlock solution (Beyotime, P0222) for 10 min to avoid the endogenous antigen. The sections were incubated with anti‐LC3 (Abcam, ab192890), anti‐Sec31a (Santa Cruz, sc‐376587), anti‐Sec24a (Proteintech, 15958‐1‐AP) and anti‐Osx (Santa Cruz, sc‐393325) for 14 h, then stained with fluorescence‐labeled secondary antibodies Alexa Fluor 488 (Abcam, ab150113), Alexa Fluor 594 (Abcam, ab150080) and Alexa Fluor 647 (Abcam, ab150115). The nucleus was highlighted with DAPI (Abcam, ab104139). The pictures were captured with the confocal microscopy (Olympus, FV‐3000), and analyzed by the software ImageJ. Cells were grown on 14 mm coverslips to semiconfluency. The samples were fixed with 4% paraformaldehyde for 20 min at room temperature, permeabilized with 0.1% Triton X‐100 (Beyotime, P0096) for 15 min and then blocked with 5% BSA for 30 min at room temperature. The primary antibody was incubated with the samples at 4 °C for 16–18 h. After three washes, cells were incubated with a diluted Alexa Fluor‐conjugated secondary antibody in 5% BSA in PBS plus 0.1% Tween‐20 at room temperature for 1 h. The nuclei were highlighted with DAPI (Boster, AR1176) at room temperature for 10 min. The primary antibodies for SEC31a (Santa Cruz Biotechnology, sc‐376587), SEC24a (Santa Cruz Biotechnology, sc‐517155, ATG9a (Abcam, ab108338), p62 (ABclonal, A19700), LAMP2 (ABclonal, A1961), WIPI2 (Proteintech, 28820‐1‐AP), and FIP200 (Proteintech, 17250‐1‐AP), were used. Corresponding fluorescent secondary antibodies were applied. Images were obtained with a confocal microscope (Olympus, FV3000). The Coloc 2 plugin was utilized to obtain Spearman's coefficient using the Fiji software v2.3.0.

### Statistical Analysis

The quantitative data were presented as mean ± SD for at least three independent experiments using GraphPad Prism 7. An unpaired t‐test was used for comparing two groups with one variance, and the one‐way ANOVA test was employed for comparing multiple groups with more than one variance followed by equal variance as determined Bonferroni multiple comparisons post hoc test. Significance was defined as ^*^ *P* < 0.05, ^**^ *P* < 0.01 and ^***^ *P* < 0.001.

## Conflict of Interest

The authors declare no conflict of interest.

## Author Contributions

J.N. and S.M. contributed equally to this work. J.N., S.M., and D.P. performed experiments, analyzed, interpreted the results and co‐wrote the manuscript. L.W., Y.L. and J.C. performed experiments. M.L., P.M., P.C. and A.L. provided critical scientific input and/or reagents. D.P. conceived, designed and funded the study, analyzed and interpreted the results, and wrote the manuscript. All authors contributed to the final review and editing of the paper.

## Supporting information

Supporting Information

## Data Availability

The data that support the findings of this study are available on request from the corresponding author. The data are not publicly available due to privacy or ethical restrictions.
