# Supplementary material for: SEC31a‐ATG9a Interaction Mediates the Recruitment of COPII Vesicles for Autophagosome Formation
Source: Adv Sci (Weinh). 2024 Oct 3;11(44):2405127. doi: 10.1002/advs.202405127 (PMC11600210; doi:10.1002/advs.202405127)
Supplement: Supplementary file 1 — Supporting Information [file ADVS-11-2405127-s001.docx]

Supporting Information

SEC31a-ATG9a Interaction Mediates the Recruitment of COPII Vesicles for Autophagosome Formation

Jiaming Nie, Shaoyang Ma, Linyue Wu, Ye Li, Jiao Cao, Meng Li, Peter Mei, Paul R. Cooper, Ang Li and Dandan Pei*


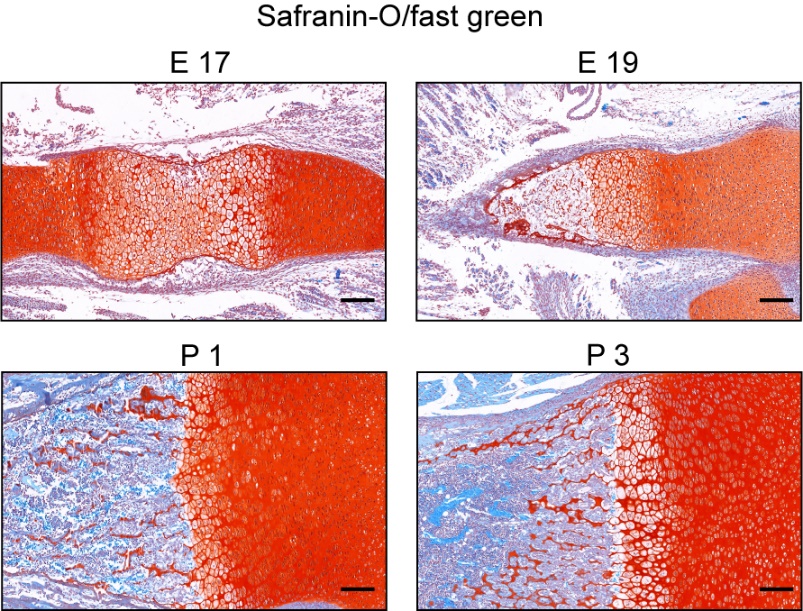


Figure S1 Safranin-O/fast green staining of the right femur of rats at E 17, E 19, P 1 and P 3. Scale bars: 100 μm.


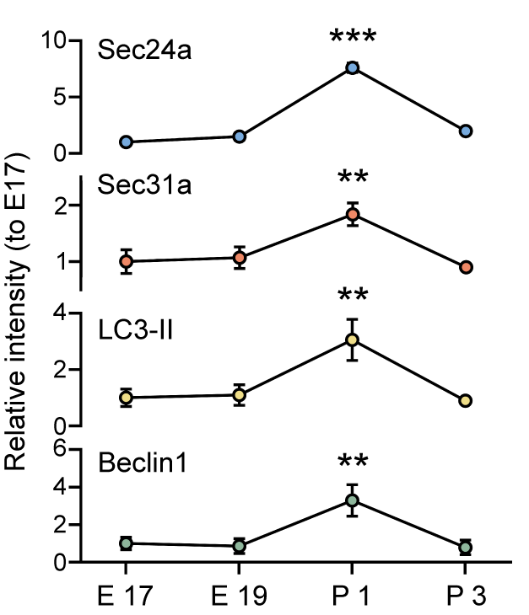


Figure S2 Quantification of the expressions of Sec24a, Sec31a, LC3-II and Beclin1 results with time. E 17: embryonic day 17, E 19: embryonic day 19, P 1: postnatal day 1, P 3: postnatal day 3.


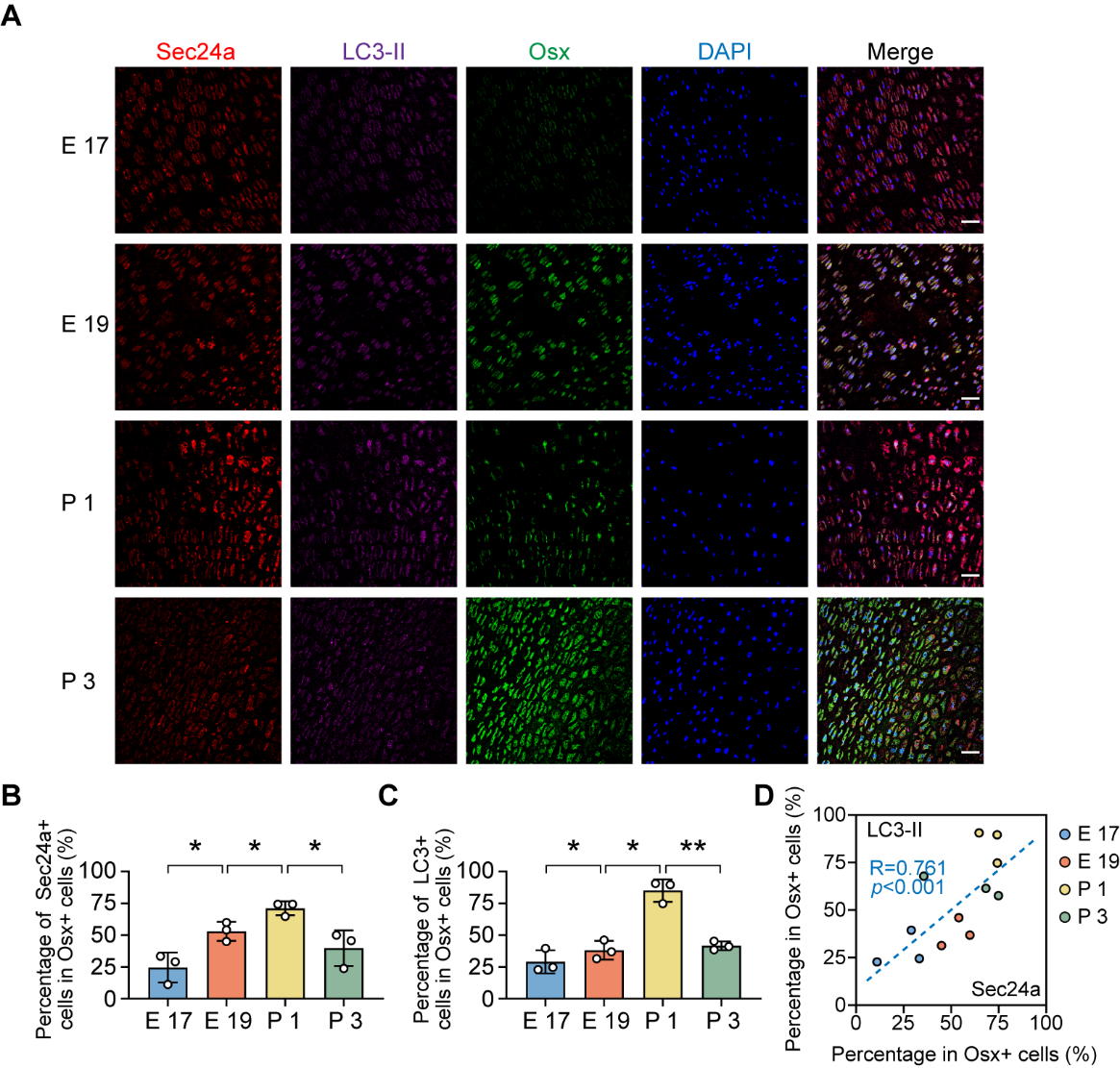


Figure S3 Three-color immunofluorescence (Sec24a, LC3-II and Osx) staining results of rat femurs. (A) Three-color immunofluorescence staining for Sec24a (Red), Osx (Green) and LC3-II (Purple) expression of femurs from E 17, E 19, P 1 and P 3 rats, and quantitative immunofluorescence analysis of Sec24a (B) and LC3-II (C) in Osx+ cells. Scale bar: 20 μm (left) and 10 μm (right). (D) The correlation between Sec24a-LC3-II was indicated based on the immunofluorescence staining intensity. E 17: embryonic day 17, E 19: embryonic day 19, P 1: postnatal day 1, P 3: postnatal day 3. * *P* < 0.05; ** *P* < 0.01.


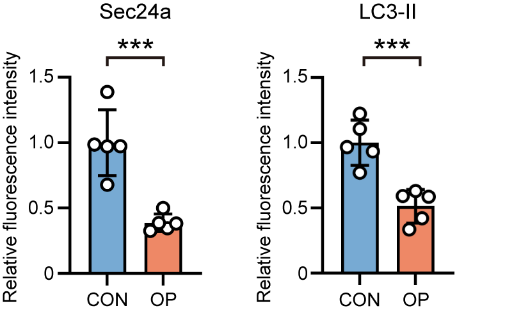


Figure S4 Quantitative immunofluorescence analysis of Sec24a and LC3-II in femur from rats with or without osteoporosis. *** *P* < 0.001.


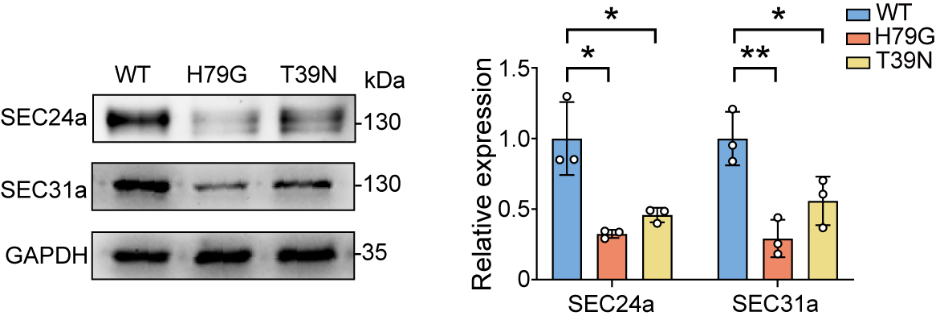


Figure S5 WB analysis and quantification showed the effect of disruption of COPII vesicles on the expressions of SEC24a and SEC31a.


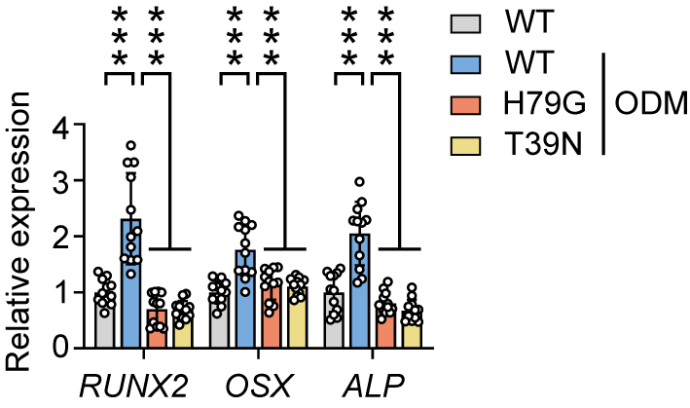


Figure S6 RT-qPCR analysis of *RUNX2*, *OSX* and *ALP* expressions in BMSCs transfected with H79G or T39N vector.


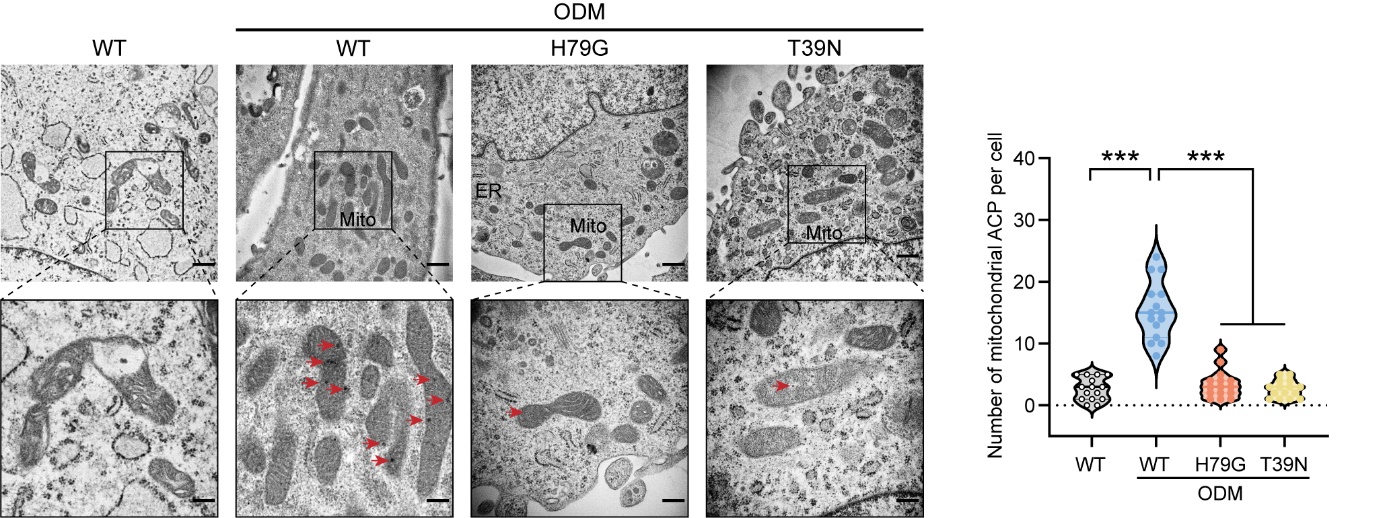


Figure S7 TEM shows nanoparticles of amorphous calcium phosphate (ACP) in BMSCs under osteogenic induction. Scale bars: 2 μm. White arrows indicate the ACP in mitochondria. Scale bars: 1 μm. * *P* < 0.05, ** *P* < 0.01, *** *P* < 0.001 (two-tailed t-test).


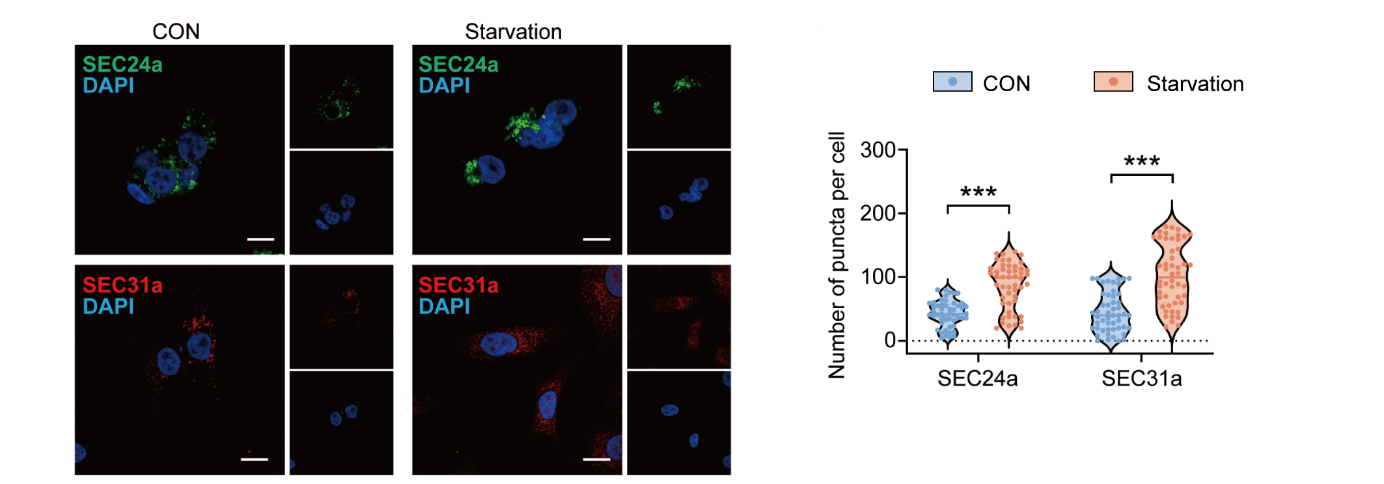


Figure S8 Immunofluorescence of SEC24a and SEC31a of BMSCs under starvation condition. Scale bars: 20 μm. Quantification of the immunofluorescence signals of SEC24a and SEC31a from BMSCs in under starvation condition.


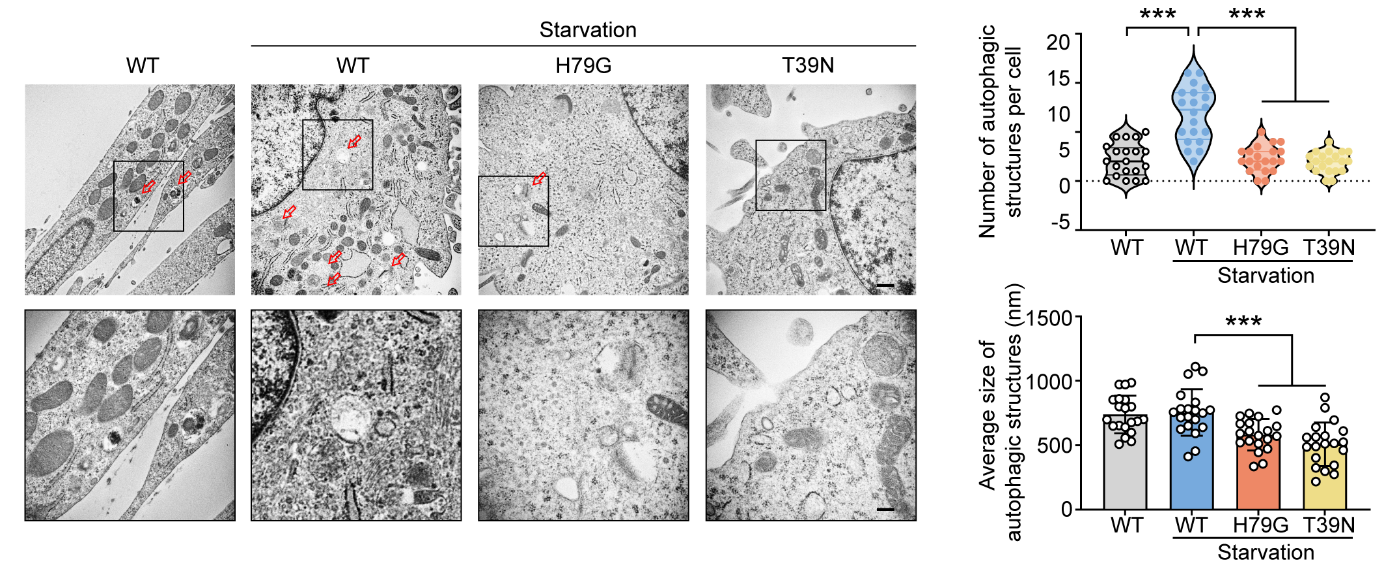


Figure S9 The number of autophagosomes in the BMSCs transfected with H79G or T39N vector was detected by transmission electron microscopy (TEM). Scale bars: 2 μm. Red fingers illustrated typical autophagosomes. Scale bars: 1 μm. Quantification of the numbers of autophagic structures in BMSCs transfected with H79G or T39N vector under starvation condition. Quantification of average size of autophagic structure in BMSCs transfected with H79G or T39N vector under starvation condition. *** *P* < 0.001 (two-tailed t-test).


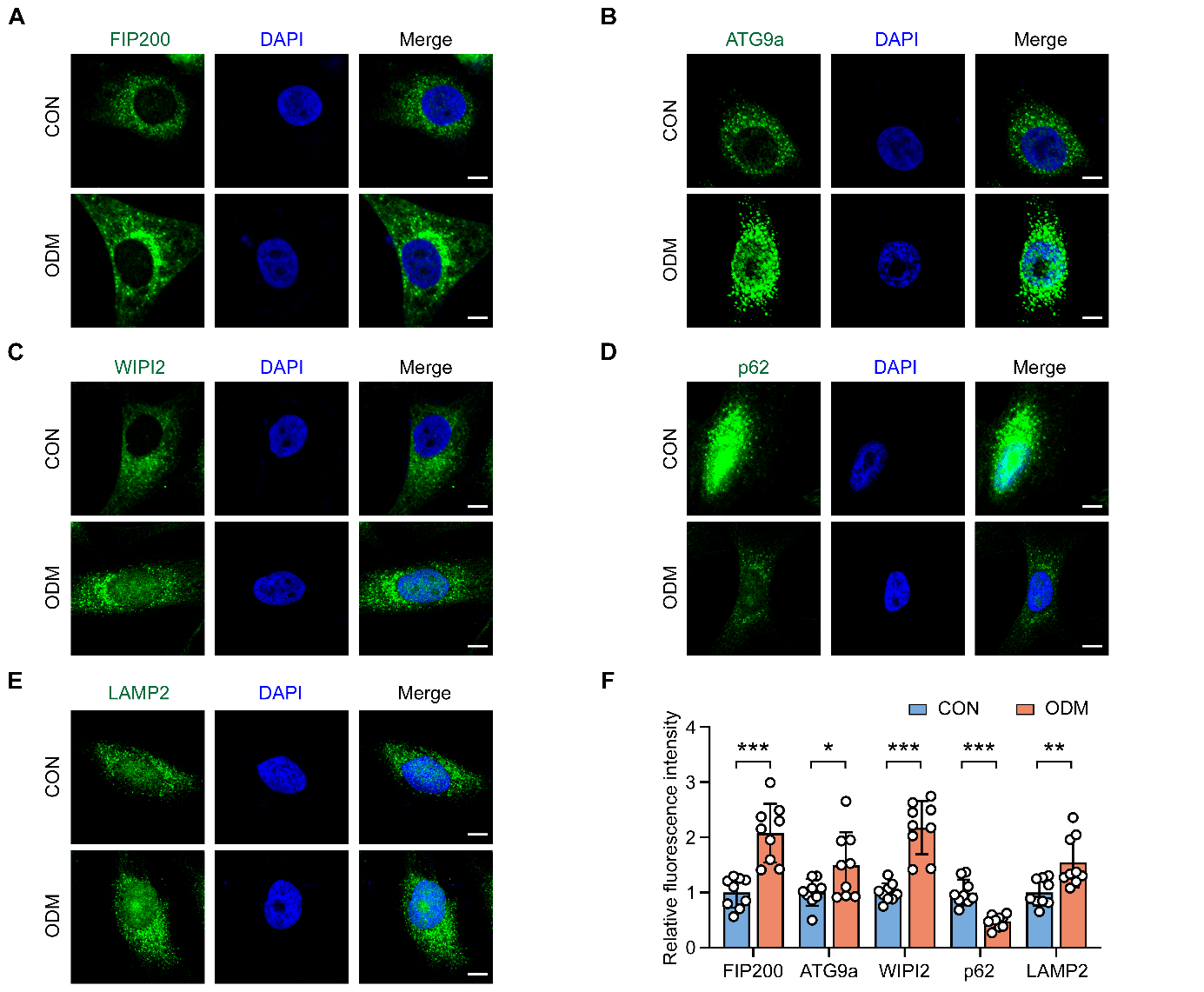


Figure S10 COPII vesicles function at the early stage of autophagosome formation. (A-E) Fluorescence confocal microscopy revealed the expression of FIP200 (A), ATG9a (B), WIPI2 (C), p62 (D) and LAMP2 (E) in BMSCs under osteogenic induction. Scale bars: 10 μm. (F) Quantitation of autophagy-related protein expressions in BMSCs under osteogenic induction. * *P* < 0.05, ** *P* < 0.01, *** *P* < 0.001 (two-tailed t-test).


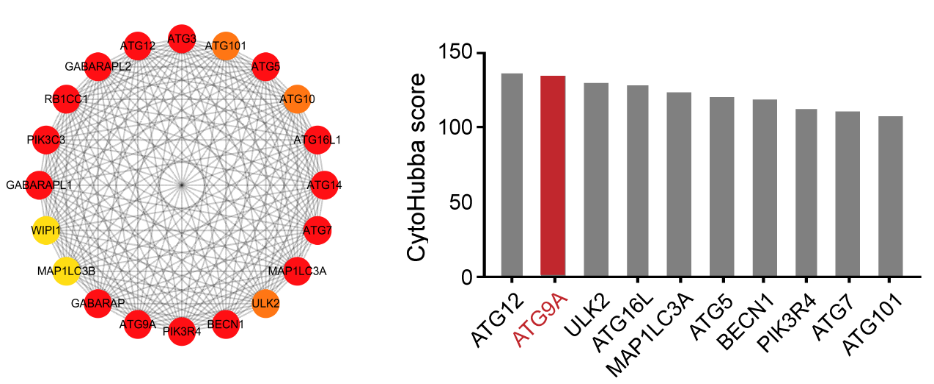


Figure S11 The cytoHubba analysis of autophagy-related proteins.


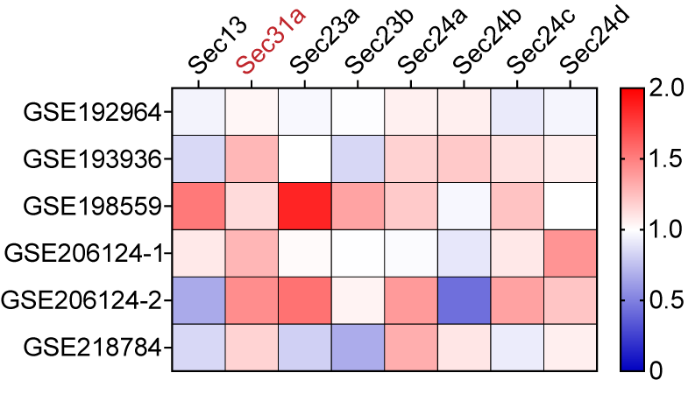


Figure S12 The publicly available data derived from GEO database shows the expression of COPII vesicle in mineralization. Heatmap colors represent the relative expression of each gene ranging from 0 (blue) to 2.0 (red).


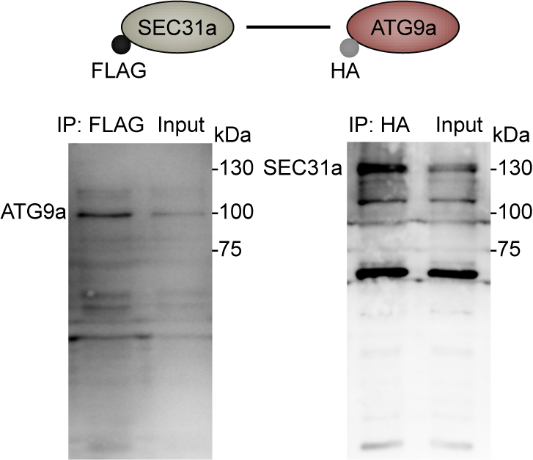


Figure S13 Ectopically expressed ATG9a interacts with SEC31a. Flag‐SEC31a was overexpressed in HEK293T cells together with the individual HA‐tagged ATG9a as indicated. Proteins were purified by IP with Flag or HA beads, following Western blot to detect ATG9a or SEC31a with an HA antibody or Flag antibody.


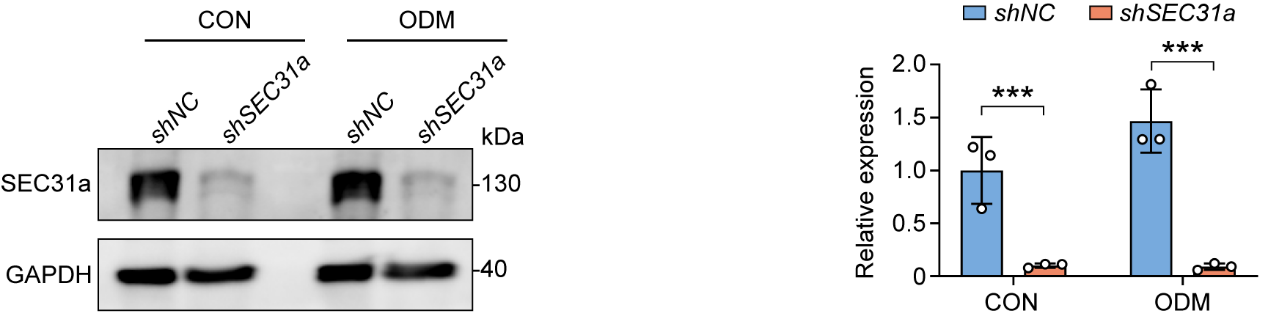


Figure S14 Interference of SEC31a restrains autophagy and osteogenesis in BMSCs. WB analysis and quantification showed the effect of sh-SEC31a on the expression of SEC31a protein of BMSCs under osteogenic induction.


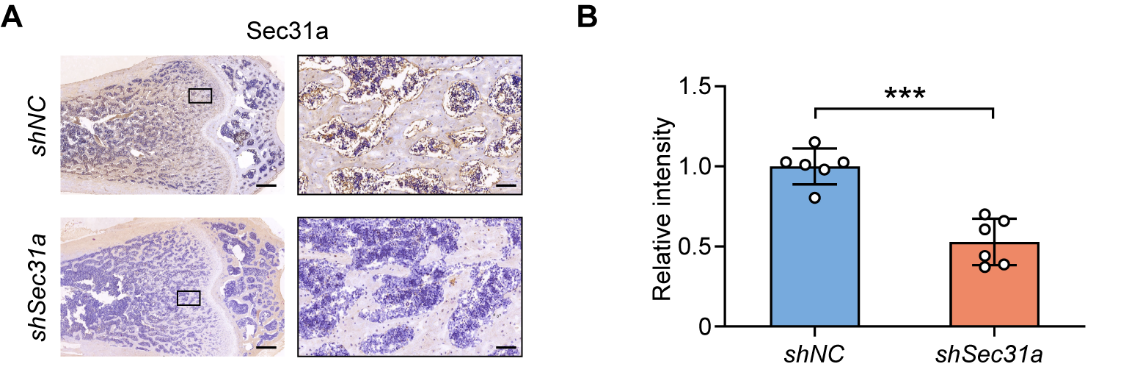


Figure S15 Injection of *sh-Sec31a* adenovirus into bone marrow cavity suppresses the mineralization of rat bone tissue. (A) Immunohistochemical staining of SEC31a in rats bone tissue. Scale bars: 200 μm and 20 μm, respectively. (B) Quantitative analysis of immunohistochemical staining intensity. *** *P* < 0.001 (two-tailed t-test).


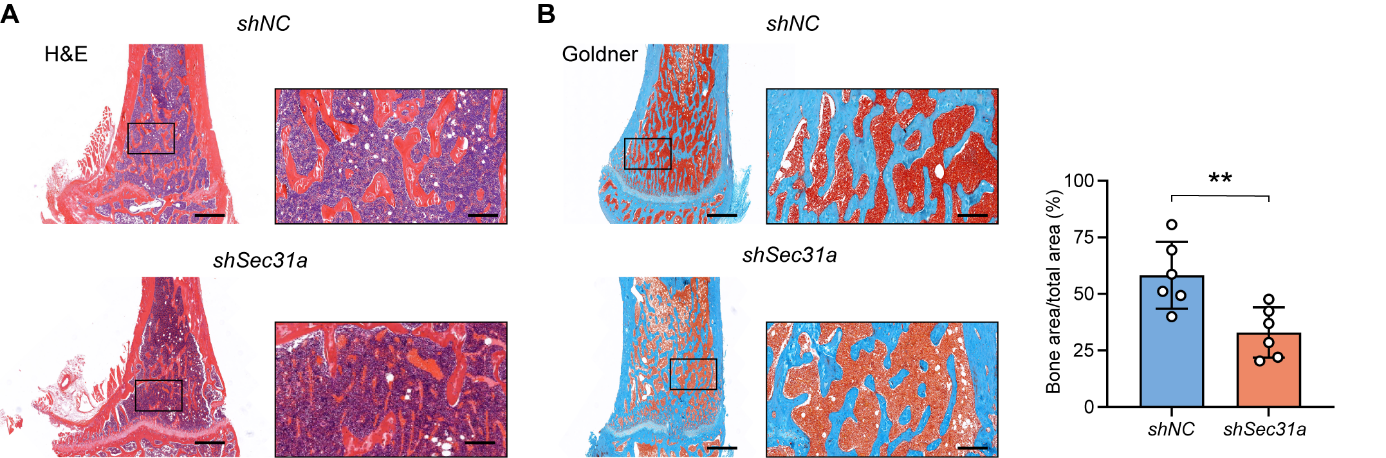


Figure S16 Representative images of H&E (A) and Goldner (B) staining of the femur of rats with or without injection of *sh-Sec31a* adenovirus. The quantification of the ratio of bone area to total area was shown on the right. Scale bars: 200 μm (left) and 50 μm (right).
